# Gene expression profile of peripheral blood mononuclear cells in mild to moderate obesity in dogs

**DOI:** 10.1016/j.vas.2021.100183

**Published:** 2021-06-18

**Authors:** Sayaka Miyai, Amin Omar Hendawy, Kan Sato

**Affiliations:** a Department of Animal Health Technology, Yamazaki University of Animal Health Technology, Hachioji, Tokyo, Japan; b Department of Biological Production, Tokyo University of Agriculture and Technology, Fuchu, Tokyo, Japan; c Department of Animal and Poultry Production, Damanhour University, Damanhour, Egypt; d Laboratory of Animal Nutrition, Division of Life Science, Graduate School of Agricultural Science, Tohoku University, Sendai, Miyagi, Japan

**Keywords:** Obesity, PBMC, Transcriptomes, Biomarker, Hemoglobin subunits

## Abstract

**Background:**

Molecular mechanisms and early diagnosis on the development of mild to moderate of canine obesity are not understood although recent dog obesity is a widespread problem. To understand the differences between normal weight and mild to moderate obesity, the purpose of this study is to investigate the gene expression profiles of peripheral blood mononuclear cells (PBMC) in dogs.

**Methods:**

This study comprised a sample of 12 privately-owned *Miniature Dachshund,* which were divided into two groups (obese and control) based on body condition scores (BCS). Serum biochemical parameters and PBMC gene expression profiles were compared between groups.

**Results:**

A statistically significant between group differences was recorded for body weight (BW), BCS, serum Insulin and triglyceride (TG) levels (*p* < 0.05). RNA-seq revealed the upregulated 154 genes and the downregulated 198 genes in obese dogs at more than 3.5-fold change compared with control animals. Hemoglobin subunits alpha- and beta-like were detected in the downregulated genes. RT-PCR analysis showed downregulation of *FOLH1, ALAS2* and *LOC100855540* genes, and upregulation of *BCL2L15* gene, suggesting that the metabolic difference between normal and mild to moderate obesity was involved in the hemoglobin metabolism.

**Conclusions:**

This study revealed significant differences in the gene expression of *BCL2L15, FOLH1, ALAS2*, and hemoglobin subunits such as *LOC100855540* between normal weight and mild to moderate obese dogs, which indicate that these genes may prevent the obesity in dogs and be potentially useful for diagnosis of mild to moderate obesity.

## 1 Introduction

Obesity is associated with several forms of cancer, chronic conditions such as cardiovascular diseases and type 2 diabetes as well as several musculoskeletal disorders, and has an enormous direct impact on quality of life (Visscher & Seidell, 2001). Recently, dog obesity is a widespread problem and the prevalence of obesity and overweight in dogs in the Western countries has been reported to be almost 50%, as in human (Grant, Vester Boler, Ridge, Graves, & Swanson, 2011; Switonski & Mankowska; 2013; Usui, Yasuda, & Koketsu, 2016; Wallis, Szabó, Erdélyi-Belle, & Kubinyi, 2018). Several papers have reported some breeds such as Beagle, Dachshund and Retrievers seemed to be predisposed to obesity and in particular, Small dogs such as *Miniature Dachshunds* have been reported as having the highest risk for being overweight and obesity in Japan (Switonski & Mankowska; 2013; Usui et al., 2016). Canine obesity has also brought about veterinary concerns such as diabetes mellitus, cardiovascular disease, dyslipidemia and orthopedic disorder, which has serious effects on morbidity and mortality (Grant et al., 2011; Usui et al., 2016). Purina PetCare research have proven that dog's median life span can be extended by restricting diet to maintain ideal body condition (Kealy et al., 2002). Therefore, veterinary hospital and owners are requiring remedies to treat and prevent the occurrence of obesity-related complicated disorders in mild to moderate obesity.

Obesity results when energy intake exceeds energy expenditure and adipocyte occur hypertrophic and hyperplastic expansion owing to overfeeding, which energy in the form of triglyceride (TG) is stored in it (Iyer et al., 2019; Mizunoe et al., 2019). Adipocytes account for the majority of White adipose tissue (WAT) and it also function as endocrine organ that secretes adiponectin, leptin, and pro-inflammatory cytokines such as tumor necrosis factor-*α* (TNF-*α*), interleukine-1*β* (IL-1*β*), and interleukine-6 (IL-6). Besides, those cytokines released from dysfunctional adipocytes promote to secrete C-reactive protein (CRP), consequently, which contribute to systemic low-grade inflammation (Grant et al., 2011; Kawasumi et al., 2018; Mizunoe et al., 2019). In obese animal, *β*-oxidation of fatty acids in mitochondria is markedly activated in various tissues and excess amount of reactive oxygen species (ROS) is produced (Kawasumi et al., 2018). However, observations derived from these pure lineages in rodent may not be similar to those in the human and canis, since obesity is known to be a multifactorial disease (Fuchs et al., 2018).

PBMC are mainly composed lymphocytes and monocytes which circulate around the body, which can be easily obtained from blood samples without performing biopsy (Jung, Seo, Ryu, & Choi, 2016). PBMC gene expression profiles correlate with adipose, muscle and liver tissue gene profiles, which reflect carbohydrate, lipid metabolism and immune response in obesity-associated organs because they are exposed to both environmental factors and metabolic tissues (Jung et al., 2016; Larsen et al., 2018). Several canine studies have investigated to attempt to identify transcriptional profile that distinguish predisposing factors in disease, metabolism and nutrition from a healthy state with using PBMC (Anderson et al., 2018; Hulanicka, Garncarz, Parzeniecka-Jaworska, & Jank, 2014; Schnurr, Reynolds, Komac, Duffy, & Dunlap, 2015). Few transcriptome studies of gene expression of adipose or muscle tissues in canine obesity have shown relation of endocannabinoid metabolism, insulin signaling, oxidative stress, mitochondrial homeostasis and extracellular matrix (Grant et al., 2011; Grant, Vester Boler, Ridge, Graves, & Swanson, 2013a; Iyer et al., 2019). However, the biological processes involved in canine obesity are not fully clarified. In, addition, transcriptional biomarkers of PBMC associated with the metabolic differences between normal and obese was not certainly identified, even though the canine models were used to study human heart diseases, which are associated with obesity (Camacho, Fan, Liu, & He, 2016). Therefore, the purpose of this study was to determine the gene expression profiles in normal weight and obese dogs using RNA-seq analysis and to identify the possible molecular mechanisms underlying the development of canine obesity.

## 2 Materials and method

### 2.1 Ethics statement

This study was performed in accordance with the ethical guidelines of the Yamazaki Gakuen University (Current Yamazaki University of animal Health Technology), and it was approved by Animal Experimentation Committee (approval number: H29YUAE No.1 and 10).

### 2.2 Animals

Data of the current study were collected from privately-owned *Miniature Dachshund* reared at individual environments (*n* = 12, age: 5.75±0.60). Animals were divided into 2 groups (control and obese). Each group consisted of 6 dogs based on body condition scores (BCS) and body weight. The BCS for each dog was clinically assessed by inspection and palpation, and scores were recorded on 5-point scale system (1, thin; 2, underweight; 3, normal; 4, overweight; 5, obese) (Usui et al., 2016). Animals of a BCS score of 3 were allocated to the control group, whereas animals with a BCS score of 4–5 were allocated to the obese group. Complete blood counts (CBC) in both groups were within reference range and until now they also had no problems in veterinary health history. The gender in control group was consisted of male 1, female 1, cast 1 and spay 3. On the other hand, those in obesity group were consisted of female 2 and cast 4.

### 2.3 Analyses of serum biochemicals

Venous blood samples were collected in silica gel tubes (BD, USA) after overnight fasting (≧ 12 h) once per dog. Blood samples were centrifuged at a speed of 1200 RCF (g). Serum levels of glucose (GLU), triglyceride (TG), total cholesterol (T-cho), alanine transaminase (ALT), aspartate transaminase (AST) and C-reactive protein (CRP) were measured by using a Fuji DRI-CHEM dry chemistry analyzer (DRI-CHEM 4000 V, Fujifilm, Tokyo). Serum Leptin and Insulin levels were estimated by using ELISA kit (Merck, USA and Mercodia, Sweden).

### 2.4 RNA isolation, RNA-seq analysis and real-time RT-PCR

**Fig. 1:**
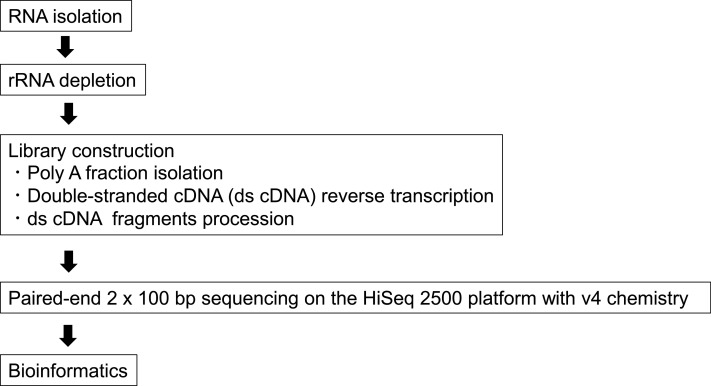
The workflow from RNA isolation to functional annotation of genes.

PBMC were isolated from EDTA-treated blood samples after overnight fasting (≧12 h) by density-gradient sedimentation of Ficoll-method with Histopaque reagent-1077 (Sigma-Aldrich, USA) and used for total RNA extraction. Total RNA was extracted using TRIzol regent (Thermo Fisher scientific, USA) according to the manufacturer's instructions. Purified total RNA samples of both groups were analyzed by RNA-seq method (eurofins genomics, Japan). Upregulated and downregulated genes significantly differentially expressed based on a false-discovery rate (FDR) < 5%, Probability (*P*) value < 0.05 and fold change > 3.5 compared with control group were chosen. RNA quality was determined with the Agilent 2100 Bioanalyzer (Agilent Technologies, Massy, France) and only the samples with an RNA integrity number (RIN) > 8.5 were further used for RNA-sequencing, and quantitative real-time PCR. RNA-Seq libraries were prepared and sequenced at Hokkaido system science (Sapporo, Japan). A TruSeq Stranded mRNA kit (Illumina, San Diego, CA) was used to prepare libraries of the RNA-Seq from total RNA samples extracted from PBMC of overweight dogs (*n* = 6) and normal dogs (*n* = 6). Libraries were prepared according to the manufacturer's protocol. Briefly, the sequence kit was used SBS/Cluster Kit v2 and the sequencing run was performed with HiSeq 2500 (Illumina, San Diego, CA), in single-end mode. Illumina BaseSpace-created FASTQ files were used for further analysis (Rapid Run). Workflow from RNA isolation to functional annotation of genes showed Fig. 1

To confirm and evaluate RNA-seq data, total RNA isolated from each sample was amplified by RT-PCR method (Takara, Japan) to obtain complementary DNA (cDNA). Functional and interesting 9 genes (*NDUFV3, BCL2L15, NRCAM, LOC6511565 LOC1008555400, ALAS2, FOLH1, PLAT* and *NTRK2*) were chosen and analyzed independently by using TB Green premix Ex Taq II and Thermal Cycler Dice Real time system II (TAKARA, Japan). Primer concentration used at 0.2 μM. Melting curve in all products was confirmed only one curve at 95°C (melt temperature: 95°C), and those were absence of primer dimers. Results were normalized with *RSP9* in reference gene and compared to the control group.

### 2.5 Kyoto encyclopedia of genes and genomes (KEGG) analysis of the differentially expressed genes

To investigate the functional associations of upregulated and down regulated genes in obese group compared with control group, we performed a Gene Ontology (GO) and KEGG pathway using the DAVID (the Database for Annotation, Visualization, and Integrated Discovery). The heatmap was created in MeV version 4.9.

### 2.6 Statistical analysis

All experimental data are shown as means±standard error of the mean (SEM). Biochemical data were analyzed with unpaired Student's *t*-test or Mann-Whitney's *U* test in Statcel 4 (The Publisher OMS Ltd., Japan). Significance was considered at a p value of 0.05.

## 3 Results

### 3.1 Body weight, body condition scores and serum biochemicals

**Fig. 2:**
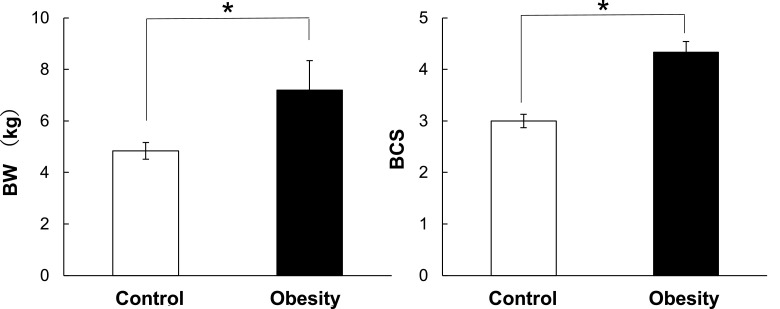
BW and BCS in the obese and control groups. Data are represented means±standard error (SE), *n* = 6, *p < 0.05.

Fig. 2 illustrates between group differences in BW and BCS. Both BW and BCS of obese dogs were significantly higher than those in the control group (*p*  < 0.05).(Table 1)

**Table 1:** Forward and reverse primers used for RT-PCR.

**Gene symbol**	**Primer(5′→3′)**		**Amplicon size**	**Gene ID**
*NRCAM*	F	CAACTCGACAAAAGCGTTCA	174	475881
	R	CACATAAGGCGACAGCTTCA		
*LOC611565*	F	TTGGCTTGCATATGTTGTTTGTG	248	611565
	R	CACAGATGACCTGGGCAGTAAGT		
*NDUFV3*	F	AGTTCCTCAGGAGAGCAAAGGAA	151	478421
	R	CATCATCTGAGGATCAACCTTGG		
*BCL2L15*	F	AGTTCCTCAGGAGAGCAAAGGAA	151	606767
	R	CATCATCTGAGGATCAACCTTGG		
*LOC100855540*	F	ACTTCAAGCTCCTGAGCCACTG	142	1.01E+08
	R	GCAGCTTAACGGTACTTGGAGGT		
*ALAS2*	F	CAGAGAAGGTCACACACCTGGTT	216	491498
	R	CATTACTGCACCAGACTGACACG		
*PLAT*	F	ATGCAACTGTGACCAAGGACATT	120	482840
	R	TACACTGTGTGATGGCTCAGCTC		
*FOLH1*	F	TTCAATCCCAGTGGAAGGAATTT	194	476775
	R	AGGTGGCACAACATCTGAAACAT		
*NTRK2*	F	ATTGGAATGACCAAGATCCCTGT	231	484147
	R	GTCACTGGCATCCTTCAGTGTCT		

Although serum GLU, T-cho, AST, ALT, CRP and Leptin concentrations did not vary between obese and control groups, levels of Insulin and TG were significantly higher in obese animals compared with normal weight control counterparts (Table 2).

**Table 2:** Serum levels of glucose metabolism (GLU and Insulin), lipid metabolism (TG and T-cho), liver function markers (AST and ALT), inflammation marker (CRP) and adipokine (Leptin) in the obese and control groups.

	**Control**	**Obesity**
GLU (mg/dL)	97.8 ± 4.18	92.7 ± 6.11
Insulin (mU/L)	7.05 ± 1.36	14.5 ± 2.96*
TG (mg/dL)	58.8 ± 4.22	102 ± 16.2*
T-cho (mg/dL)	148 ± 8.01	196 ± 23.8
AST (U/L)	31.3 ± 1.98	28.7 ± 2.11
ALT(U/L)	47.0 ± 3.89	43.8 ± 10.0
CRP (mg/dL)	0.92 ± 0.08	1.08 ± 0.19
Leptin (ng/mL)	2.47 ± 1.08	7.25 ± 2.26

Data are represented by means ± standard error (SE), (*n* = 6), *p < 0.05.

GLU: glucose, TG: triglyceride, T-cho: total cholesterol, AST: aspartate transaminase, ALT: alanine transaminase, CRP: C-reactive protein

### 3.2 Gene expression profiles of PBMC by RNA-seq and RT-PCR

**Fig. 3:**
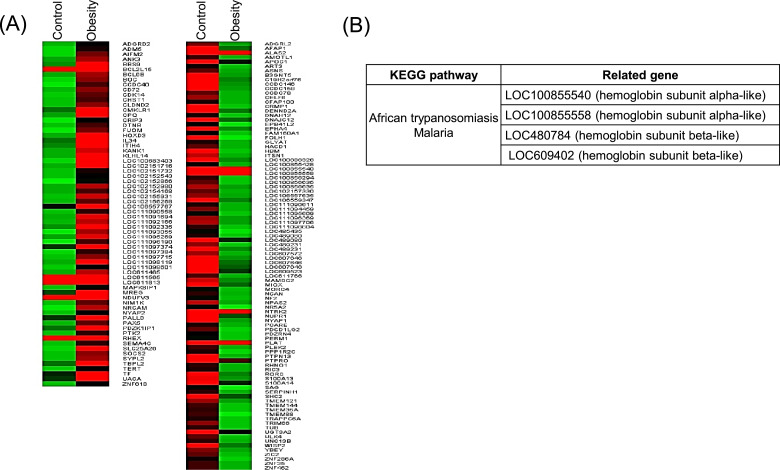
Differential expression of genes in obese and control groups. (A) Heatmap of PBMC differential gene expression profiles in obese and control groups. The level of expression of each gene in each sample relative to the median level of expression of that gene across all samples is represented using a red, black and green color scale (green-below median; black-equal to median; red-above median). (B) The KEGG pathway and associated gene using David analysis in downregulated genes in the obese group.

RNA-seq identified genes that were differently expressed in the obese group compared with those in the control group. In all, 154 genes were up-regulated, and 198 genes were down-regulated in obese dogs at fold change > 3.5, respectively, compared with those in the control group. Fig. 3A shows the heatmap of PBMC gene expression profiles in control and obesity group at more than 3.5-fold change. To further evaluate the functional differences in PBMC transcriptomes of dogs in the obese group compared with those in the control group, we performed David analysis using genes that were differentially expressed (fold change > 3.5) compared with those in the control group. These results are indicated in Fig. 3B. Although no signaling pathway was detected in upregulated genes, the African trypanosomiasis and Malaria pathways, as well as the expression of certain genes such as hemoglobin subunit alpha- or beta- like were noticed in downregulated genes.

The typical functional 4 up-regulated genes (*NDUFV3*: 5.1 fold change, *LOC611565*: 4.4 fold change, *BCL2L15*: 4.2 fold change and *NRCAM*: 8.2 fold change) were chosen, and 5 down-regulated genes (*FOLH1*: -25 fold change, *LOC100855540* (hemoglobin subunit alpha like): -5.9 fold change, *ALAS2*: -5.6 fold change, *NTRK2*: -4.9 fold change and *PLAT*: -4.3 fold change) in the obese group (Table 3).

**Table 3:** Upregulated genes (*n* = 4) and downregulated genes (*n* = 5) in the obese group compared with the control group in RNA-seq.

**Up-regulated genes**	**Fold change**	**Down-regulated genes**	**Fold change**
*NDUFV3*	5.1	*FOLH1*	−25
*LOC611565*	4.4	*LOC100855540*	−5.9
*BCL2L15*	4.2	*ALAS2*	−5.6
*NRCAM*	8.2	*NTRK2*	−4.9
*/*		*PLAT*	−4.3

These genes were selected based on FDR < 5%, *P* values < 0.05 and > 3.5-fold change.

*NDUFV3* encodes a NADH dehydrogenase [ubiquinone] flavoprotein 3, *LOC611565* encodes antigen WC1.1-like, *BCL2L15* encodes a BCL2 family kin (Bfk) isoform b, *NRCAM* encodes a neuronal cell adhesion molecule, *FOLH1* encodes a folate hydrolase 1, *LOC100855540* encodes a globin domain-containing protein, *ALAS2* encodes a 5′-Aminolevulinate Synthase 2, *NTRK2* encodes a neurotrophic receptor tyrosine kinase 2, *PLAT* encodes a tissue-type plasminogen activator. *LOC100855540* and *ALAS2* are genes which related to hemoglobin.

**Fig. 4:**
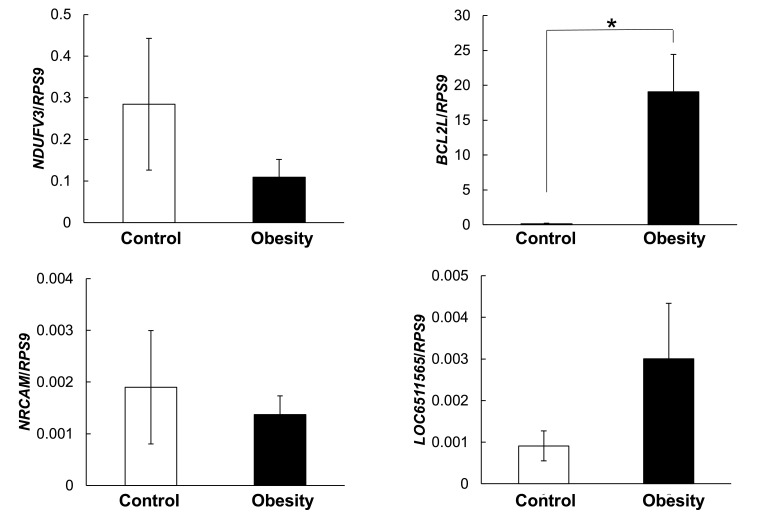
Validation of RNA-seq upregulated genes by real-time RT-PCR. Data are represented by means ± standard error (SE), (*n* = 6), *p < 0.05 **p < 0.01. □: control group, ■: obese group. Results were normalized with *RSP9* (40S ribosomal protein S9).

**Fig. 5:**
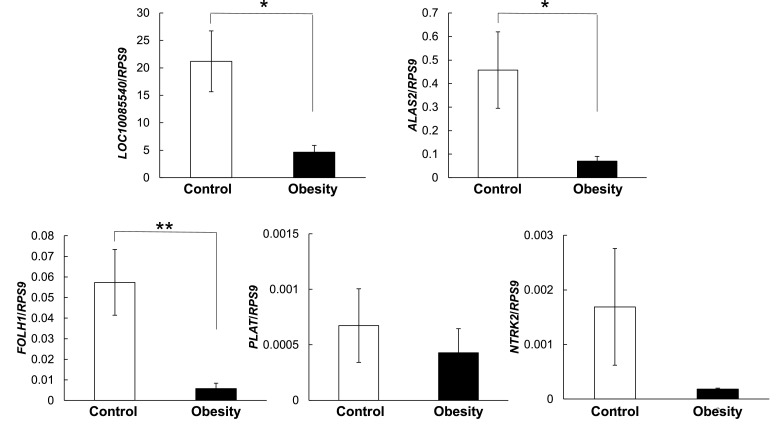
Validation of RNA-seq downregulated genes by real-time RT-PCR. Data are represented by means ± standard error (SE), (*n* = 6), *p < 0.05 **p < 0.01. □: control group, ■: obese group. Results were normalized with *RSP9* (40S ribosomal protein S9).

Expressions of these selected genes were confirmed by RT-PCR (Fig. 4, Fig. 5).

There were significant differences in the gene expression of *BCL2L15, FOLH1, LOC100855540* and *ALAS2*, while no differences were found for the gene expression of *NDUFV3, LOC611565, NRCAM, NTRK2* and *PLAT* by real-time RT-PCR.

## 4 Discussion

The characterization of gene profile of PBMC in mild to moderate obesity in dogs have revealed novel details about the specific features of the expression of important molecules in lipid metabolism. The PBMC expresses the novel components identified as essential for prevention of obesity, and those are related to the hemoglobin metabolism, such as *LOC100855540* and *ALAS2*.

To clarify the differences of gene expression profiles in normal and obese, we first categorized the “mild to moderate of obesity” in dogs. Plasma biochemical parameter of obese dogs was characterized the increases of GLU, TG, T-cho, AST, ALT, Insulin, CRP, and Leptin concentrations as compared to normal dogs. The present study revealed that there were no differences in the biochemical parameters, except for TG and Insulin concentrations, between control and obese dogs (Table 2). In contrast, BW and BCS in obese group were significantly higher than those in control group (Fig. 2). Leptin concentration in obese group may be higher than that in control group (Table 2) since Leptin concentration is known to positively correlate with BCS (Ishioka et al., 2007). These results suggest that animals in the obese group may be in a mild to moderate of obesity given that serum concentrations of all parameters rise in later-stages of obesity (Grant et al., 2011; Grant et al., 2013a; (Ishioka et al., 2007); Kabir et al., 2015; Kawasumi et al., 2018). Then, the dogs were appropriately categorized to two groups to identify transcriptional biomarkers in PBMC that may distinguish predisposing factors of obesity-associated diseases from a healthy state.

A novel finding of this study was revealed by the RNA-seq and additional RT-PCR analysis of the PBMC as indicated by a significant downregulation of African trypanosomiasis or Malaria pathway in obese group (Fig. 3B), that is novel details about the mild to moderate of obesity in dogs. In African trypanosomiasis and Malaria pathways, hemoglobin subunit alpha- or beta- like and enzyme of rate-limiting step during heme biosynthesis were noticed in downregulated genes. Hemoglobin subunit alpha like *LOC100855540* gene is highly homologous to that of hemoglobin subunit alpha (HBA) in human. Hemoglobin is expressed in both erythroid cells and a variety of none-erythroid cells. In erythroid cells, two alpha globin chains assemble with two beta globin chains to form adult hemoglobin heterotetramers with four heme prosthetic groups, which carry oxygen to tissues and transport carbon dioxide away from tissues to the lungs (Sangwung et al., 2017). Some studies have shown that murine mutations that affect alpha- and beta- chains can change the affinity of hemoglobin for oxygen and the related efficiency of oxygen utilization (Yang et al., 2018). Hence, down-regulation of *LOC100855540* (Fig. 5) may limit the transport of oxygen in the circulation. Nevertheless, low expression of hemoglobin alpha- or beta- chain in obese dogs is not directly related to those parasite diseases (African trypanosomiasis or Malaria).

5-aminolevulinic acid synthase 2 (*ALAS2*), which catalyzes the initial and rate-limiting step during heme biosynthesis, is an erythroid-specific mitochondrial gene. The expression of *ALAS2* during erythroid differentiation is strongly activated to meet the demand for hemoglobin. A former study demonstrated that *ALAS2* mutation causes severe anemia mechanistically by arresting erythroid differentiation at the proerythroblast stage due to heme insufficiency (Liu et al., 2018). Therefore, down-regulation of *ALAS2* (Fig. 5) may inhibit heme biosynthesis and limit circulation of oxygen, yet little is known about how canine obesity affects *ALAS2*. Furthermore, recent research has indicated obese dogs have a low arterial partial pressure of oxygen (PaO_2_), short inspiratory time and expiratory time, and a high respiratory rate, compared with dogs with an ideal BCS (Pereira-Neto et al., 2018). It is intuitive that changes in hemoglobin affinity for oxygen during mild to moderate obesity in canine would not be as serious as in anemia since hematocrit and hemoglobin concentrations did not significantly vary in obesity and control groups. However, slight changes in hemoglobin affinity for oxygen may affect mitochondrial energy metabolism. Numerous studies have reported hypoxia induces an increase in erythropoietin (EPO) hormone production in the kidney, which then circulates in the plasma and binds to receptors abundantly expressed on erythroid progenitor cells (Bunn, 2013), thereby promoting the differentiation of erythroid precursors and increasing red blood cell mass. The oxygen-carrying capacity of the blood is thereby enhanced, increasing tissue oxygen tension, this completing the feedback loop and suppressing further expression of EPO. As mentioned above, slight reductions of O_2_ are suggested to promote the production of EPO and maintain red blood cell mass by compensating for the downregulation of genes (*LOC100855540* and *ALAS*2) and reducing its function.

Another study in obese human has shown to be reduced mitochondrial oxidative capacity (oxygen consumption rates) of adipocytes and mitochondria biogenesis, which has occurred metabolic alterations, insulin resistance, and low-grade inflammation (Bhatraju & Agrawal, 2017; de Mello, Costa, Engel, & Rezin, 2018). The obesity induced-reactive oxygen species (ROS) and oxidative stress can overwhelm the Tricarboxylic acid cycle (TCA) cycle and mitochondrial respiratory chain causing a mitochondrial dysfunction (de Mello et al., 2018). Hence, any change of the mitochondrial-related genetic expressions may have negative effect on hematopoiesis in bone marrow and levels of oxygen circulation in the body. Similarly, transcriptome study using adipose tissue in canine obesity found that alteration of mitochondrial homeostasis (Grant et al., 2011). It is not clear why hemoglobin-associated genes were detected from PBMC, however, several studies have also shown diet induced obesity results in a altering hematopoietic stem and progenitor cells in bone marrow, which adipogenesis is also tightly associated with angiogenesis in early stage (Ambrosi et al., 2017; Emmons, Niemiro, & De Lisio, 2017; Grant, Vester Boler, Ridge, Graves, & Swanson, 2013b). Thus, our data in hemoglobin-associated genes may reflect on the adipogenesis effect in bone marrow or other adipocyte, and inhibit hematopoietic system.

In current study, the expression of *BCL2L15* and *FOLH1* in the mild to moderate obesity groups were shown in the significant changes compared to those in control group (Figs 4 and 5), whereas those genes were not belonged with the specific metabolic pathway in PBMC prepared form obese dogs. The *BCL2L15* is a gene that encodes BCL2 family kin (Bfk) isoform b. BCL protein family includes members that contain both pro- and anti-apoptotic molecules. Thus, they regulate cell death by controlling mitochondrial outer membrane permeabilization. Overexpression of Bfk is known to promote weak apoptosis activity and antagonize the anti-apoptosis function of BCL2 (Ban, Tozaki, & Nakano, 2016; Coultas et al., 2003; Dempsey et al., 2005; Gurung, Lukens, & Kanneganti, 2015; Ozören, Inohara, & Núñez, 2009; Pujianto et al., 2007). Human Bfk also needs to be cleaved by caspase to become a pro-apoptotic protein, which suggests that it acts as an amplifier of the apoptotic signal rather than an initiator (Gurung et al., 2015). Therefore, the noticed up-regulation of *BCL2L15* in obese dogs in this study (Fig. 4) signify that this gene may play a major role in decreasing mitochondrial membrane potential and promoting the release of cytochrome c, which enhances apoptosis.

The *FOLH1* (folate hydrolase 1), known as glutamate carboxypeptidase II (GCPII), is a gene that codes for enzymes predominantly involved in the hydrolysis of dietary polyglutamyl folates by sequential cleaving terminal γ-linked glutamate residues from dietary polyglutamyl folates. Dietary folates are composed of a mixture of monoglutamyl and polygulutamyl forms that are hydrolyzed to the monoglutamyl form prior to transport across the jejunal brush border membrane. Evidence indicates that poor intestinal absorption of folate due to downregulation of *FOLH1* may lead to relative hypercysteinemia and alteration of the metabolism of glucose and lipid (Šilhavý et al., 2018). Folate is an essential cofactor in metabolic pathways that influence DNA methylation patterns, DNA synthesis and cell proliferation (Martino et al., 2018). A recent study shows that low levels of serum folate are frequently associated with obesity whereas chronic consumption of high-fat diets impairs folate transporter expression and leads to diminution of hepatic folate storage (Sid, Siow, Shang, & Woo, 2018). Taken together, down-regulation of *FOLH1* may contribute to elevation of glucose and lipid concentrations beyond reference ranges in the future mainly through affecting folate transporter expression or hepatic folate storage. However, we did not find the elevation of glucose and lipid concentrations, and also the differential gene expressions of glucose and lipid metabolism in present study. Although our findings revealed no differences in red blood cell numbers, hematocrit value and hemoglobin concentration between both groups (data not shown), inactivity of folate in obese dogs may slightly have negative effects on DNA synthesis and apoptosis in erythroblasts. This notion may be supported by another main finding of the current study: down-regulation of *LOC100855540* in obese dogs (Fig. 3, 5 and Table 3).

Some studies have reported gene expression between PBMC and tissues are conflicting since some genes are expressed in tissue-specific manner, consequently, it is necessary to access to biological material (Afman et al., 2012; Brattbakk et al., 2013; de Mello, Kolehmanien, Schwab, Pulkkinen, & Uusitupa, 2012; Pinhel et al., 2017). Meanwhile, other studies suggest that the gene expression in PBMC reflect the glucose or lipid metabolism and inflammation in obesity-associated organ (Jung et al., 2016). Our study revealed physiological changes and identified transcriptional biomarkers from PBMC in obese dogs compared with control dogs although it would be necessary to compare its genes between PBMC and specific tissue. Several canine studies have attempted to detect the differential gene expression between lean and obese dogs with using skeletal muscle or adipose tissue and had enrichment insulin signaling and hematopoietic cell lineage pathway in obese dogs (Grant, 2013a and b), however, our study have aimed to analyze the differences among healthy state and mild to moderate obesity. Moreover, we provided useful information about potential blood-based targets for preventing or treating obesity-associated complications by using not experimental dogs but companion dogs.

In conclusion, we examined metabolic changes and gene expression profiles of PBMC according to BCS. The dogs of mild to moderate obesity demonstrated significant differences in only TG and Insulin in reference range compared with dogs with normal weight in the control group, although the other parameters were no differences. In present condition, RNA-seq and RT-PCR analyses in PBMC revealed upregulation of genes possibly associated with proapoptotic function in obese dogs. Moreover, the expression of genes involved in hemoglobin subunit, heme biosynthesis in erythroid mitochondria and the hydrolyzing folates was downregulated in the PBMC of obese dogs. Thus, our present study signifies that these genes can be potentially useful for the diagnosis of early obesity. However, the present study was provided the evidence under the small sample size and limited conditions such as gender, and then further studies are necessary in order to elucidate the role of these genes in mechanism of obesity.

## Funding

No funding was received for this work.

## Ethical statement for solid state ionics

Hereby, I / Sayaka Miyai / consciously assure that for the manuscript / Gene expression profile of peripheral blood mononuclear cells in primary-stage obesity in dogs / the following is fulfilled:

1) This material is the authors' own original work, which has not been previously published elsewhere.
2) The paper is not currently being considered for publication elsewhere.
3) The paper reflects the authors' own research and analysis in a truthful and complete manner.
4) The paper properly credits the meaningful contributions of co-authors and co-researchers.
5) The results are appropriately placed in the context of prior and existing research.
6) All sources used are properly disclosed (correct citation). Literally copying of text must be indicated as such by using quotation marks and giving proper reference.
7) All authors have been personally and actively involved in substantial work leading to the paper, and will take public responsibility for its content. The violation of the Ethical Statement rules may result in severe consequences. To verify originality, your article may be checked by the originality detection software iThenticate. See also http://www.elsevier.comfeditors/plagdetect.

I agree with the above statements and declare that this submission follows the policies of Solid State Ionics as outlined in the Guide for Authors and in the Ethical Statement.

## Declaration of Competing Interest

No conflict of interest exists. We wish to confirm that there are no known conflicts of interest associated with this publication and there has been no significant financial support for this work that could have influenced its outcome.
